# Taxanes enhance trastuzumab-mediated ADCC on tumor cells through NKG2D-mediated NK cell recognition

**DOI:** 10.18632/oncotarget.6353

**Published:** 2015-11-21

**Authors:** Martina Di Modica, Lucia Sfondrini, Viola Regondi, Stefania Varchetta, Barbara Oliviero, Gabriella Mariani, Giulia Valeria Bianchi, Daniele Generali, Andrea Balsari, Tiziana Triulzi, Elda Tagliabue

**Affiliations:** ^1^ Molecular Targeting Unit, Department of Experimental Oncology and Molecular Medicine, Fondazione IRCCS Istituto Nazionale dei Tumori, Milan, Italy; ^2^ Dipartimento di Scienze Biomediche per la Salute, Università degli Studi di Milano, Milan, Italy; ^3^ Department of Infectious Diseases, Fondazione Istituto di Ricovero e Cura a Carattere Scientifico Policlinico San Matteo, Pavia, Italy; ^4^ Department of Medical Oncology, Fondazione IRCCS Istituto Nazionale dei Tumori, Milan, Italy; ^5^ Dipartimento di Terapia Molecolare e Farmacogenomica, Istituti Ospitalieri di Cremona, Cremona, Italy

**Keywords:** breast cancer, docetaxel, ADCC, NK cell, NKG2D

## Abstract

Recent clinical data indicate a synergistic therapeutic effect between trastuzumab and taxanes in neoadjuvantly treated HER2-positive breast cancer (BC) patients. In HER2+ BC experimental models and patients, we investigated whether this synergy depends on the ability of drug-induced stress to improve NK cell effectiveness and thus trastuzumab-mediated ADCC. HER2+ BC cell lines BT474 and MDAMB361 treated with docetaxel showed up-modulation of NK activator ligands both *in vitro* and *in vivo*, accompanied by a 15–40% increase in *in vitro* trastuzumab-mediated ADCC; antibodies blocking the NKG2D receptor significantly reduced this enhancement. NKG2D receptor expression was increased by docetaxel treatment in circulating and splenic NK cells from mice xenografted with tumor cells, an increase related to expansion of the CD11b^+^Ly6G^+^ cell population. Accordingly, NK cells derived from HER2+ BC patients after treatment with taxane-containing therapy expressed higher levels of NKG2D receptor than before treatment. Moreover, plasma obtained from these patients recapitulated the modulation of NKG2D on healthy donors' NK cells, improving their trastuzumab-mediated activity *in vitro*. This enhancement occurred mainly using plasma from patients with low NKG2D basal expression. Our results indicate that taxanes increase tumor susceptibility to ADCC by acting on tumor and NK cells, and suggest that taxanes concomitantly administered with trastuzumab could maximize the antibody effect, especially in patients with low basal immune effector cytotoxic activity.

## INTRODUCTION

Trastuzumab, a recombinant humanized monoclonal antibody directed to the extracellular domain of the HER2 protein, is the paradigm of tailored treatment for patients with HER2-positive breast cancer. Recent clinical data indicated a synergistic therapeutic effect between trastuzumab and chemotherapy in the neoadjuvant setting in these patients, with up to 70% pathological complete response [1] and especially when taxanes are used [2;3]. Moreover, data from the NCCTG N9831 trial suggest that trastuzumab is more effective if used concurrently with rather than following the taxane component of adjuvant chemotherapy [4]. However, the mechanism(s) underlying this synergy remains unclear, prompting investigations to further define trastuzumab killing activity when administered with chemotherapy.

Several preclinical studies have indicated that trastuzumab anti-tumor activity depends on several direct or indirect cytostatic and cytotoxic activities [5;6]. The importance of immune cells in trastuzumab therapy is supported by studies showing that ADCC contributes mainly to the antitumor effects of trastuzumab in HER2-positive tumors in mice [7] and in breast carcinoma pre-operative clinical studies, in which it was associated with increased tumor infiltration of natural killer (NK) cells [8;9]. NK cells play a key role in trastuzumab-mediated ADCC [10] and, if stimulated, enhance trastuzumab efficacy [11]. The NK cell detection system, in addition to the low-affinity Fc receptor CD16, which permits NK cells to kill antibody-coated target cells, includes a variety of cell surface activating and inhibitory receptors, the engagement of which by ligands expressed on tumor cells regulates immune effector cell activities. A key receptor for NK cell activation is NK Group 2 member D (NKG2D), a type II transmembrane C-type lectin-like receptor expressed on all NK cells. It binds multiple ligands, including MHC class I chain-related A (MICA), MICB and several UL-16 binding proteins (ULBPs), which are induced after cellular stress [12] and are the most common ligands for NK cell receptors in breast carcinomas together with DNAM-1 ligands [13].

In this study, we investigated in HER2-positive breast carcinoma models the effect of taxane treatment on expression levels of ADCC-associated factors with a focus on NKG2D ligands, and correlated the changes in these molecules with the ability of trastuzumab to mediate ADCC. Moreover, we investigated the effect of chemotherapy on NK cells in xenografted mice and in HER2-positive breast carcinoma patients treated with neoadjuvant therapy.

## RESULTS

### Modulation of NKG2D ligand cell surface expression in response to docetaxel

**Figure 1:**
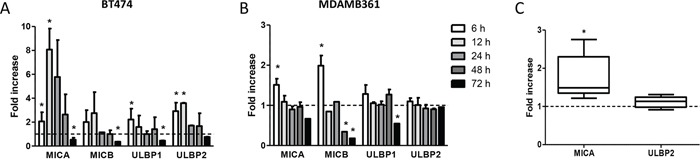
Modulation of NKG2D ligands on breast carcinoma cells in response to docetaxel treatment **A, B.** BT474 **(A)** and MDAMB361 **(B)** cells were treated with 100 nM docetaxel for the indicated times and analyzed by flow cytometry. Shown are fold-increases of ligand expression in treated versus untreated cells at the same time points. Data are mean ± SEM (*n* = 3). **C.** Fold-increase in MICA and ULBP2 protein expression levels, as assessed by Western blot and quantified by densitometric analysis using Quantity One software, in MDAMB361 breast carcinoma cells grown in SCID mice and treated with 20 mg/Kg docetaxel versus untreated tumors. Data are mean ± SEM (*n* = 5). *p < 0.05 by paired Student's *t*-test.

To investigate whether docetaxel upregulates the production of the NK cell stimulatory ligands MICA, MICB, ULBP1 and ULBP2 of the NKG2D receptor, HER2-overexpressing human breast carcinoma cell lines BT474 and MDAMB361 were treated *in vitro* for different times with 100 nM docetaxel and analyzed by flow cytometry. Docetaxel-treated cells revealed a significant increase in membrane-associated ligand expression as a rapid and dynamic event, with the greatest enhancement within 6–12 hours and a return to basal levels within 24–48 hours (Figure 1A, 1B). Longer drug treatment increased the soluble forms of MICA and ULBP2, the two molecules reportedly cleaved and released into the extracellular space as negative feedback ligand-mediated NK regulation [14], in culture medium of breast carcinoma cells at 48 and 72 hours after docetaxel treatment compared to untreated cells (Supplementary Figure S1), partly explaining their reduction on the cell membrane. Specifically, soluble ULBP2 amounts increased in both cell lines as compared to untreated cells. Similar results were obtained for soluble MICA in BT474 but not in MDAMB361 culture medium, where soluble MICA was never detectable.

To test whether NK cell stimulatory ligands are also up-modulated by docetaxel *in vivo*, SCID mice bearing MDAMB361 tumors in the mammary fat pad were treated intravenously with 20 mg/Kg docetaxel or not treated and analyzed 24 hours later for expression of MICA and ULBP2 by Western blotting on cells obtained from disaggregated tumors. Levels of MICA ligand were significantly higher in tumors derived from docetaxel-treated than untreated mice (Figure 1C and Supplementary Figure S2).

### NKG2D ligand expression and susceptibility to trastuzumab-mediated ADCC

**Figure 2:**
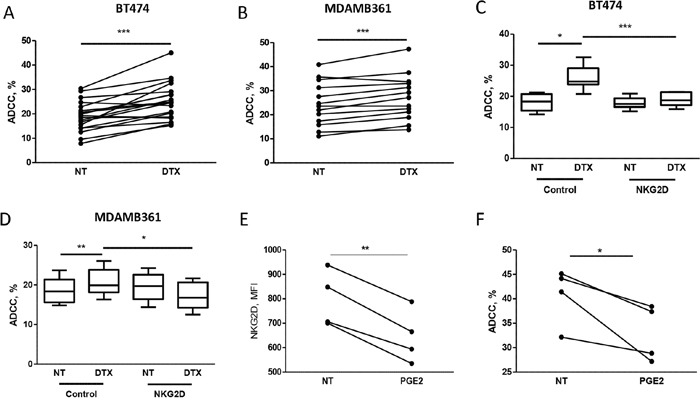
Docetaxel treatment increases trastuzumab-mediated cell cytotoxicity on tumor cells, as assessed by ^51^Cr release **A, B.** To evaluate ADCC, ^51^Cr-labeled breast cancer cell lines BT474 **(A)** and MDAMB361 **(B)** were treated for 6 hours with 100 nM docetaxel (DTX) or not treated (NT) and cultured for 4 hours with PBMCs from healthy donor blood samples in medium containing trastuzumab (4 μg/ml). Shown are percentages of lysis of target cells at 50:1 effector:target cell ratio of PBMCs from independent healthy donors (A: *n* = 19, *p* = 0.0004; B: *n* = 13, *p* = 0.0006). **C, D.** BT474 and MDAMB361 cells, respectively, treated with DTX or not treated were cultured as above with PBMCs pre-incubated for 30 minutes with blocking NKG2D blocking antibodies (1 μg/ml). Values are median, interquartile range (box), minimum and maximum. (C: *n* = 6; D: *n* = 6). **E, F.** PBMCs from independent healthy donors (*n* = 4) were treated with 100 nM PGE2 for 24 hours, analyzed by flow cytometry for NKG2D expression (MFI on NK cells, E) and used in ADCC assay (F) against BT474 cells as described above. *p < 0.05, **p < 0.01, ***p < 0.001 by paired Student's *t*-test.

To test whether increased expression of NKG2D ligands observed on breast carcinoma cells after treatment with docetaxel enhances trastuzumab-mediated cell cytotoxity, tumor cells were analyzed in ^51^Cr-release assays using human PBMCs from healthy donors as effector cells. Docetaxel treatment for 6 hours rendered BT474 and MDAMB361 target cells more sensitive to trastuzumab-dependent ADCC, with an average killing increase of 38% in BT474 (Figure 2A) and 14% in MDAMB361 (Figure 2B) cells. This ADCC increment was significantly inhibited in both cell lines by antibodies blocking the NKG2D receptor (Figure 2C, 2D), supporting the role of the NKG2D ligand-receptor interaction in increasing trastuzumab-mediated ADCC upon chemotherapy. Comparable results were obtained in BT474 target cells treated with docetaxel for 12 hours, whereas a trend toward inhibition of trastuzumab-mediated killing was found in BT474 and MDAMB361 cells treated with docetaxel for 72 hours as compared with untreated controls (Supplementary Figure S3), when NKG2D ligand expression was lower in treated than in untreated cells (Figure 1).

To further demonstrate the direct role of NKG2D in trastuzumab-mediated ADCC of HER2 positive breast carcinoma cells, PBMCs from healthy donors were collected, treated with prostaglandin-E2 (PGE2) for 24 hours to force NKG2D down-modulation [13], and used in ADCC assay against BT474 cells. The induced reduction of NKG2D expression on NK cells (Figure 2E) led to a reduced killing of BT474 cells in the presence of trastuzumab compared to untreated PBMCs (Figure 2F).

### Increased expression of NKG2D in circulating NK cells in mice receiving chemotherapy

**Figure 3:**
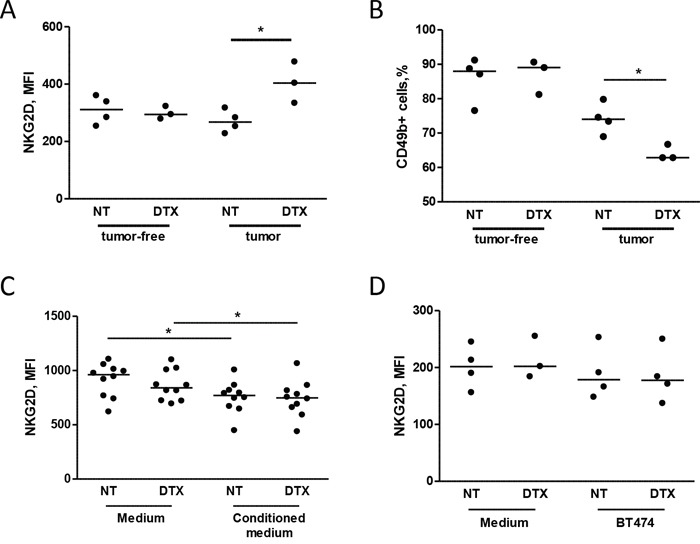
Chemotherapy alters NKG2D expression in circulating NK cells in mice injected with tumor cells **A, B.** SCID mice were xenotransplanted with MDAMB361 breast carcinoma cells and, when tumors reached a volume of 150 mm^3^, treated with 20 mg/Kg docetaxel (DTX) or not treated. Six days after treatment, NKG2D expression (MFI of NK cells, A) and NK cell proportions **(B)** were analyzed in mouse blood. The same analysis was performed in tumor-free SCID mice of the same age (A, B) *p < 0.05 by unpaired Student's *t*-test. **C, D.** PBMCs from independent healthy donors were cultured for 24 hours in medium conditioned or not by BT474 breast carcinoma cells (C) or with BT474 cells treated or not with docetaxel (D) and analyzed for NKG2D expression (MFI of NK cells) by flow cytometry. *p < 0.05 by paired Student's *t*-test.

The activity of docetaxel on effector cells was analyzed by comparing the phenotype of NK cells obtained from MDAMB361 tumor-bearing mice with that of tumor-free mice upon docetaxel treatment. No differences in NKG2D expression on NK cells were observed at 24 hours after treatment (not shown), whereas NKG2D expression was significantly higher in NK cells derived from chemotherapy-treated than saline-treated tumor-bearing mice 6 days after treatment (Figure 3A). Chemotherapy treatment impaired the proportion of NK cells only in tumor-bearing mice (Figure 3B). Notably, NKG2D expression did not differ in NK cells derived from tumor-free mice irrespective of treatment, suggesting that the presence of the tumor is necessary for up-modulation of NKG2D levels in NK cells upon chemotherapy.

To determine whether the increase in NKG2D was directly induced by tumor cells after treatment with chemotherapy, PBMCs obtained from healthy donors were cultured with supernatants conditioned by BT474 cells treated or not with docetaxel. No modulation of NKG2D expression was found in NK cells conditioned by supernatant of BT474 cells treated or not with docetaxel (Figure 3C) or co-cultured for 24 hours with BT474 cells in the presence or absence of docetaxel (Figure 3D). Similar results were observed in NK cells after 48 hours of co-culture (data not shown). These data suggest that docetaxel-induced up-modulation of the NKG2D receptor observed in mice is not directly affected by tumor cells after treatment with chemotherapy, but is likely mediated through their interaction with the host microenvironment.

**Figure 4:**
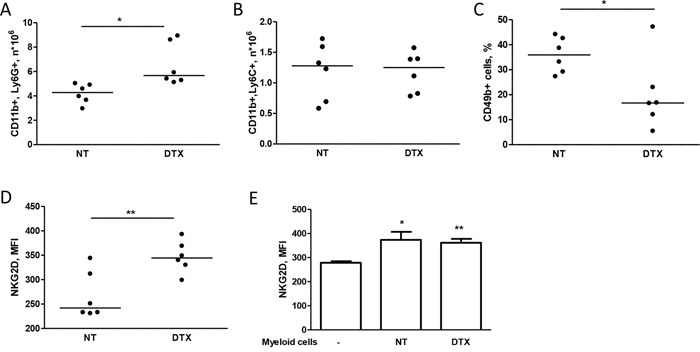
Chemotherapy alters NKG2D expression of NK cells through myeloid cells **A, B, C, D.** Mice bearing MDAMB361 tumors were treated with docetaxel (DTX) and 6 days later, spleens were analyzed for NK and myeloid cell expansion. CD11b+Ly6G+ **(A)** CD11b+Ly6C+ **(B)** NK (CD49b+) cells **(C)** and NKG2D expression (MFI of NK cells, **D)** were analyzed by flow cytometry. **E.** Splenocytes from tumor-free SCID mice were co-cultured or not with purified GR1+ myeloid cells for 24 hours and analyzed for NKG2D expression by flow cytometry (*n* = 6). *p < 0.05, **p < 0.01 by unpaired Student's *t*-test.

Based on the reported accumulation of myeloid cells in tumor-bearing mice and in cancer patients [15], we investigated the expansion of these cells in the spleen of mice injected with MDAMB361 cells and treated with docetaxel or untreated. Docetaxel treatment led to an increase in the number of CD11b^+^Ly6G^+^ cells (granulocytic myeloid cells) as compared to the number in untreated mice (Figure 4A), whereas similar numbers of CD11b^+^Ly6C^+^ cells (monocytic myeloid cells) were found in mice irrespective of treatment (Figure 4B). The percentage of CD49+ cells (NK cells) and the NKG2D expression on NK cells were lower and higher, respectively, in treated than in untreated mice both in blood (Supplementary Figure S4) and in the spleen (Figure 4C, 4D), raising the possibility that NKG2D expression in chemotherapy-treated mice is modulated by an increase in granulocytic myeloid cells that expand only in the presence of tumors. Analysis of purified myeloid cells (GR1^+^) from mice treated or not with docetaxel and co-cultured at a 1:1 ratio with splenocytes derived from tumor-free mice indicated that NKG2D relative expression on NK cells was increased, irrespective of docetaxel treatment of mice from which they were purified (Figure 4E). Thus, the higher number of myeloid cells found in mice treated with docetaxel than in untreated mice might explain the increased NKG2D expression on NK cells.

### Increased expression of NKG2D in circulating NK cells of patients receiving chemotherapy

**Figure 5:**
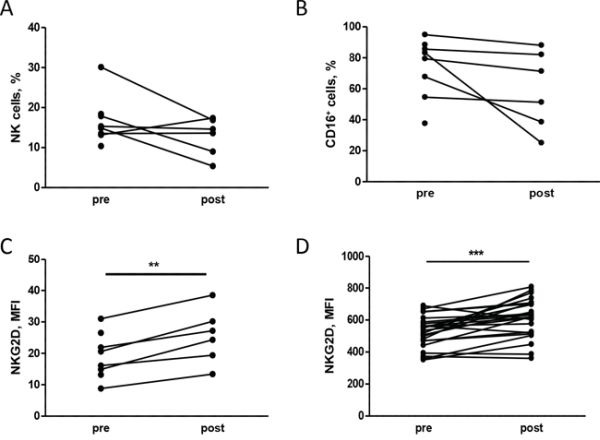
Chemotherapy alters the NK cell phenotype in human patients **A, B, C.** PBMCs isolated from patients at different time points during neoadjuvant treatment (pre: before any treatment, post: after chemotherapy) were analyzed by flow cytometry. The percentage of NK cells, identified as CD3– CD56+ **(A)** and their proportion of CD16+ **(B)** and the NKG2D expression **(C)** are shown. **D.** NKG2D expression on NK cells (CD3− CD56+ CD16+) of PBMCs from independent healthy donors treated *in vitro* with plasma derived from patients pre and post treatment. **p < 0.01, ***p < 0.001 by paired Student's *t*-test.

Based on the results obtained in preclinical models, we investigated the NK cell phenotype in patients with HER2-positive breast carcinomas treated with neoadjuvant chemotherapy and trastuzumab. Analysis of the NK phenotype in PBMCs obtained from 9 patients before (pre) and after (post) the first chemotherapy treatment cycle (4 cycles of taxane-based chemotherapy) showed that treatment slightly impaired NK cell proportions (Figure 5A), in particular CD16^+^ NK cells (Figure 5B), but significantly increased NKG2D expression on NK cells (Figure 5C). Note that other natural cytotoxicity receptors involved in human NK activity (i.e., NKp30, NKp44 and NKp46) were not significantly modified by chemotherapy (Supplementary Figure S5). Thus, chemotherapy appears to change the NK cell surface receptor configuration in patients with HER2-positive breast carcinomas. Moreover, healthy donor PBMCs cultured for 24 hours with plasma obtained from patients post-treatment showed significant up-modulation of NKG2D on NK cells compared to plasma collected pre-treatment (Figure 5D), indicating that the plasma treatment recapitulated the drug-induced up-modulation of NKG2D on NK cells.

### Increased expression of NKG2D in patients and trastuzumab-mediated ADCC

**Figure 6:**
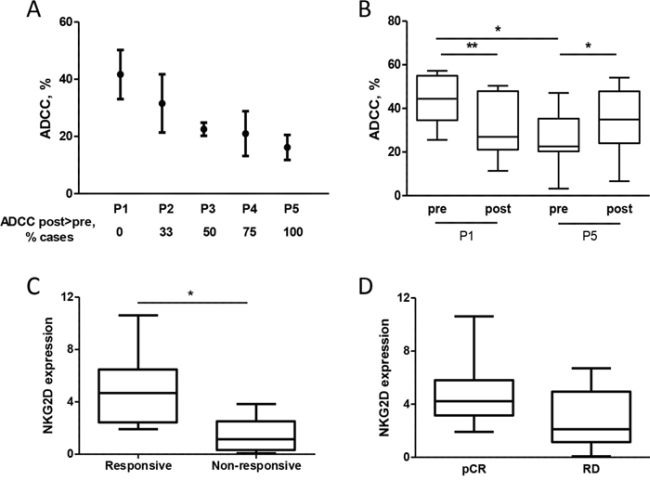
Expression of NKG2D in patients is associated with trastuzumab-mediated ADCC **A.** Trastuzumab-mediated lysis of ^51^Cr-labeled BT474 cells induced by healthy donor PBMCs (*n* = 4) treated with plasma obtained from 5 patients (P1-P5) before docetaxel administration. Data are mean and range. ADCC post > pre refers to the percentage of times that PBMCs treated with post-treatment plasma induced greater trastuzumab-mediated ADCC of BT474 cells than when treated with the pre-treatment plasma. **B.** ADCC of BT474 cells induced by PBMCs obtained from healthy donors treated with pre- and post-treatment plasma of patients P1 and P5 (*n* = 7). *p < 0.05, **p < 0.01 by paired Student's *t*-test. **C, D.** NKG2D expression as evaluated by qPCR using RNA obtained from the blood buffy-coat of 18 HER2-positive breast cancer patients before any treatment according to response to one cycle of trastuzumab alone (C) and according to pCR (D) RD: residual disease. *p < 0.05 by unpaired Student's *t*-test.

To determine whether enhanced NKG2D expression after chemotherapy is associated with improved NK cell function in the context of breast cancer, PBMCs obtained from healthy donors were treated with plasma from patients and used in ADCC assay. Treatment of PBMCs from healthy donors with pre-treatment plasma from different patients induced different ADCC ability of the same PBMC (Figure 6A), and the mean cytotoxic activity of effector cells correlated with NKG2D expression evaluated on NK cells (*r* = 0.86, *p* = 0.06). Interestingly, the lower the PBMC lytic activity induced by pre-treatment plasma, the higher the fold-increase in PBMC ADCC activity induced by post-treatment versus pre-treatment plasma (Figure 6A and Supplementary Figure S6). Indeed, treatment of PBMCs from healthy donors with patient P1 post-treatment plasma, which induced the highest expression of NKG2D on NK cells and, in turn, the highest trastuzumab-mediated ADCC before chemotherapy, did not induce a significant increment in trastuzumab-mediated ADCC compared to pre-treatment plasma (Figure 6B). By contrast, post-treatment plasma derived from patient P5 induced an increment in NKG2D expression and consequently of ADCC compared to the corresponding pre-treatment plasma (Figure 6B), which had the lowest basal activity (Figure 6A). Notably, the trastuzumab-mediated ADCC induced by NK cells after treatment with P5 post-treatment plasma increased to levels similar to those obtained with NK cells after P1 pre-treatment plasma (Figure 6B). These data suggest that the benefit of chemotherapy in improving trastuzumab-mediated ADCC occurs mainly in patients with low basal cytotoxic activity of immune effector cells, and that addition of chemotherapy to antibody administration may not be as relevant in improving trastuzumab activity for patients with elevated basal lytic activity of effector cells. Consistent with this view, NKG2D basal expression in a new series of 18 HER2-positive breast cancer patients before neoadjuvant treatment with one cycle of trastuzumab alone [16] and analyzed by qPCR using RNA obtained from the buffy-coat of collected blood was higher in tumors that benefit from the antibody, evaluated as at least 20% reduction in the standardized uptake value evaluated by FDG PET/CT scan (Figure 6C), than in non-responsive tumors (*p* = 0.0249). Moreover, patients that reached a pCR at the end of the neoadjuvant treatment with trastuzumab and docetaxel showed higher basal NKG2D expression than did partial responders with borderline statistical significance (Figure 6D, p = 0.0806); the two patients of the INT cohort with the highest NKG2D were those with a pCR after chemotherapy and trastuzumab treatment (*p* = 0.0142).

## DISCUSSION

In the present study, we report for the first time that taxanes significantly increase NKG2D ligand expression on tumor cells and their susceptibility to NK activity. Moreover, docetaxel induces the expression of the activating cognate receptor NKG2D on NK cells, increasing their cytotoxic activity mediated by trastuzumab.

The up-modulation of NKG2D ligands by chemotherapy is consistent with the function of these ligands, whose expression is generally linked to cellular stress mechanisms that induce danger signals [17]. Based on the demonstrated role of NKG2D signals in inducing NK activation favoring NK degranulation rather than adhesion to tumor cells [18] during ADCC, the induced trastuzumab activity after chemotherapy treatment observed in our model is likely due to the interaction between NKG2D ligands with their receptor, as supported by the ability of anti-NKG2D receptor antibodies to abrogate the chemotherapy-induced increase of *in vitro* trastuzumab-dependent ADCC. The involvement of the NKG2D ligand/receptor interaction in increasing trastuzumab-mediated ADCC observed here is in agreement with the increased activity of an anti-ERBB2-NKG2D ligand fusion protein targeting HER2 tumors and triggering lymphocytes that express the NKG2D receptor compared to anti-HER2 antibody alone in preclinical models [19].

Since chemotherapy is known to cause bone marrow suppression that manifests as reduced numbers of peripheral blood immune cells [20], we also evaluated its effect on ADCC effector cells. Although the percentage of NK cells was slightly reduced by chemotherapy, NKG2D expression on NK cells was increased after docetaxel treatment. While NKG2D up-modulation upon drug treatment was strictly dependent on the presence of tumor cells in mice, such cells do not appear to play a direct role in enhancing NKG2D expression, since no direct modulation of NKG2D on NK cells was observed by co-culturing PBMCs with tumor cells according to treatment with docetaxel. However, we did found that NKG2D up-modulation on NK cells after chemotherapy treatment involves the host microenvironment, specifically granulocytic myeloid cells, as indicated by the increase of NKG2D on NK cells after co-culture of GR1^+^CD11b^+^ cells with splenocytes derived from healthy mice. Indeed, neutrophils have been reported to play a role in regulating differentiation and activation of NK cells [21]. Moreover, the selective expansion of CD11b^+^Ly6G^+^and the increase in NKG2D expression on NK cells in our models upon docetaxel treatment is consistent with the elevation of granulocytic myeloid cells after chemotherapy in patients [22], and with the increase in GM-CSF and IL6 production, that supports myeloid cell expansion, as well as the increased NK cell cytotoxic activity demonstrated upon taxane treatment in human patients [23]. We speculate that tumor cells after chemotherapy treatment modulate the production of soluble factors that induce expansion of myeloid cells [24;25], as we observed in breast cancer cell lines *in vitro* after chemotherapy treatment (not shown). Myeloid cells, in turn, could sense chemotherapy and increase expression of NKG2D ligands described to induce NK activity when expressed on myeloid cells [26]. Because docetaxel is also able to induce NKG2D ligand expression in tumor cells, the interaction between tumor NKG2D ligands and NKG2D receptor expressed by NK cells significantly improves trastuzumab-mediated ADCC. Although the modulation of the NKG2D ligand/receptor could be coincidental, our findings open the door to further research aimed at harnessing the power of the modulation of both elements for improving therapy. Moreover, our results obtained regarding NKG2D raise the possibility that other activating receptors and co-regulators such as DNAM-1, described to be involved in breast cancer-NK cell recognition [13], are involved in increasing trastuzumab activity in humans and await further investigation.

Our data obtained in experimental models support the notion that chemotherapy causes modifications important for inducing an increase in trastuzumab-mediated cytotoxicity and consequent response of patients to treatment. Thus, while trastuzumab might also augment chemotherapy-induced tumor toxicity, causing attenuation of DNA repair activity and increasing apoptosis [27], our results strongly suggest that the reported synergy between trastuzumab and docetaxel in HER2-positive breast cancer patients and in nude mice bearing HER2-expressing tumors and treated with the two drugs in combination [27;28] depends on the increased ADCC rather than on the inhibition of HER2-mediated DNA repair by the antibody. Indeed, taxanes, drugs that showed the best therapeutic effect when combined with trastuzumab [2;29], presumably act as inhibitors of microtubules rather than as intercalants of the DNA and are thus unlikely to promote DNA repair mechanisms involving HER2. However, since recent evidence suggests that taxane can also induce breaks in single-strand DNA [30], it is possible that such a mechanism also contributes to the synergy between trastuzumab and taxane. The up-modulation of NKG2D in NK cells of breast carcinoma patients after treatment with taxane-containing chemotherapy further supports the relevance of drug treatment in improving the ability of trastuzumab to trigger host immune effectors against tumor cells. Thus, the evidence that taxanes significantly increase tumor susceptibility to NK activity provides an avenue to improving the cytotoxic effect of trastuzumab by injecting the antibody concomitantly with taxanes in a neo-adjuvant setting. Such a protocol could also reduce the duration of therapy.

Although based on a small subset of patients, our data suggest that the benefit of taxanes in improving trastuzumab-mediated ADCC occurs mainly in patients with low basal cytotoxic activity of their immune effector cells and with potentially low responsiveness to trastuzumab-mediated ADCC. Indeed, the meager enhancement of NKG2D after chemotherapy recorded in women with elevated basal NKG2D expression does not appear to support the relevance of chemotherapy in improving trastuzumab activity in patients with innate high lytic activity of effector cells, consistent with results obtained in the TRUP cohort that showed high expression levels of NKG2D on NK cells from patients responsive to one cycle of trastuzumab alone. Since patients with high NKG2D expression on NK cells before any treatment are also those who reached a pCR in the TRUP and the INT cohorts, it remains to be determined whether trastuzumab alone might be sufficient for a pCR in such patients or whether the addition of chemotherapy is always necessary to generate a long-lasting response. In this context, the validation of NKG2D expression levels on circulating NK cells from patients before any treatment as a predictive marker of trastuzumab response could represent a simple tool for the selection of patients who will benefit from the addition of taxanes to antibody administration.

The relevance of circulating NKG2D-positive NK cells as indicators of antibody therapy success is strengthened by results of Fisher and coworkers [31], who showed that circulating NK cells able to mediate ADCC specifically express NKG2D, but not NKp30 or NKp46 activating receptors. Activated NK cells are known to shape the adaptive immune response [32;33], described to be crucial for the complete eradication of BC upon trastuzumab treatment both in preclinical [34;35] and clinical samples [36;37]. Differences in the patients' adaptive immunity activation or recruitment could explain the variable clinical response of patients observed after treatment with trastuzumab and chemotherapy, despite the modulation of NKG2D by chemotherapy observed in all treated patients.

Together, our data indicate that the synergy between taxanes and trastuzumab occurs mainly through the NKG2D ligand/receptor-mediated activation of NK cells and mainly in those patients with low NKG2D expression on effector cells at the time of diagnosis. The understanding of the mechanism through which NKG2D levels on NK cells are increased promises to help in improving trastuzumab activity through immunotherapy combinations for those patients who do not respond to treatment.

## MATERIALS AND METHODS

### Cell lines and treatments

Human HER2-positive breast carcinoma cell lines MDAMB361 and BT474 (American Type Culture Collection) were authenticated using the Short Tandem Repeat Profiling method in our Institute facility and maintained in appropriate medium supplemented with 10% (v/v) FBS (Thermo Scientific, Waltham, MA, USA) and L-glutamine in a 5% CO_2_ humidified chamber at 37°C. Cells were treated in complete medium with 100 nM docetaxel (DOCETAXEL, Sanofi Aventis) for different times.

### Mice and treatments

Six- to 8-week-old SCID mice (Charles River, Calco, Italy) were maintained in laminar-flow rooms at constant temperature and humidity, with food and water given *ad libitum*. Mice were injected into the mammary fat pad with 5×10^6^ MDAMB361 cells in growth medium diluted 1:1 with Matrigel (BD Bioscience, San Jose, CA, USA). Experimental protocols used for animal studies were approved by the Ethics Committee for Animal Experimentation of Fondazione IRCCS Istituto Nazionale dei tumori of Milan in accordance with institutional guidelines.

Docetaxel was administered (20 mg/Kg) intravenously. Spleen and blood were collected from mice after treatment, and single-cell suspensions obtained after lysis of red blood cells were analyzed by flow cytometry. Myeloid cells were purified from total splenocytes using biotinylated anti-GR1 antibody (Miltenyi Biotec, San Diego, CA, USA) as described [38].

### Patients

Breast carcinoma core biopsies were obtained from 8 patients (INT cohort) before a treatment protocol consisting of 3 or 4 cycles of AT (adriamycin plus taxotere) followed by 4 cycles of CMF (cyclophosphamide, methotrexate and fluorouracil) and trastuzumab at Fondazione IRCCS, Istituto Nazionale dei Tumori of Milan. Blood samples of these patients were collected at two time points during neoadjuvant treatment: pre, before any treatment; post, after AT cycles.

Eighteen buffy-coats of the “trastuzumab upfront in HER2-positive locally advanced breast cancer” (TRUP) cohort deriving from the recent prospective neoadjuvant study [16] were obtained from patients before any treatment. Patients were treated with one cycle of trastuzumab alone followed by 4 cycles of taxotere and trastuzumab. Tumor dimensions on post-therapy day 21 were measured by FDG PET/CT scan. Pathological complete response (pCR) was defined as no residual invasive tumor or *in situ* carcinoma in the primary tumor and in the nodes. Human peripheral blood and tumor specimens were obtained at Fondazione IRCCS Istituto Nazionale dei Tumori of Milan and A.O. Istituti Ospitalieri di Cremona after written informed consent from patients in accordance with the Helsinki Declaration and after institutional approval from our ethics committee (Comitato Etico Indipendente, Fondazione IRCCS, Istituto Nazionale dei Tumori) for the conduct of the study.

### Western blotting

Protein fractions were extracted from tumors grown in athymic mice and analyzed by Western blotting as described [39]. Antibodies used were: anti-vinculin (Sigma-Aldrich, St. Louis, MO, USA), anti-MICA and anti-ULBP2 (R&D Systems, Minneapolis, MN, USA).

### RNA extraction and quantitative real-time PCR (qRT-PCR)

Total RNA was extracted from buffy-coats with Qiazol^®^ (Qiagen) according to the manufacturer's instructions. cDNAs were reverse-transcribed from 1 μg of total RNA in a 20-μl volume with SuperScript III (Invitrogen) using random-hexamer primers. qRT-PCR was performed using Applied Biosystem Taqman assays (CD16: Hs.04334165_m1, NKG2D: Hs.00183683_m1) on the ABI Prism 7900HT sequence detection system (Applied Biosystems, Foster City, CA, USA). NKG2D expression levels were calculated by the comparative Ct method using CD16 as reference gene and RNA obtained from a healthy donor buffy-coat as reference sample.

### Flow cytometry

MDAMB361 and BT474 cells were exposed to docetaxel (100 nM) for 6 to 72 hours or left untreated, collected and incubated at 4°C with anti-MICA, -MICB, -ULBP1, -ULBP2 (R&D Systems), followed by incubation with anti-mouse Alexa Fluor 448-conjugated reagent (Invitrogen, Waltham, MA, USA). Samples were analyzed by gating on live cells using the FACSCanto system (Becton-Dickinson, San Jose, CA, USA) and BD FACSDiva™ software (BD Bioscience).

PBMCs isolated from patients or healthy donors were analyzed using monoclonal antibodies to human antigens, including PE-anti-CD16 (3G8, BD Bioscience), PECy5-anti-CD56 (B159, BD Bioscience), APC-eFluor780-anti-CD3 (SK7, eBioscence, San Diego, CA, USA), FITC-anti-CD69 (FN50, BD Bioscience) and APC-NKG2D (ON72, Beckman Coulter, Miami, FL, USA).

Total splenocytes or purified Gr1^+^ myeloid cells from mouse spleens were analyzed using mouse monoclonal antibodies, including PE-anti-CD49b (DX5, eBioscience), APC-anti-NKG2D (CX5, eBioscience), PE-anti-CD11 (MI/70, BD Bioscience), FITC-anti-Ly6G (1A8, Mylteni Biotec) and APC-anti-Ly6C (HK1.4, eBioscience). Samples were analyzed by gating on physical parameters using the FACSCanto II system (Becton-Dickinson) and FlowJo software (Tree Star Inc, San Carlos, CA, USA).

### Cytotoxicity assays

Breast carcinoma cells were treated or not (controls) with docetaxel (100 nM) for 6, 12 or 72 hours and labeled with 100 μCi ^51^Cr (Perkin-Elmer, Waltham, MA, USA) for 1 hour at 37°C. After 3 washes with PBS-5% FBS, cells were co-incubated for 4 hours at 37°C with PBMCs isolated by density-gradient separation using Ficoll-Paque PLUS (Amersham Biosciences, Piscataway, NJ, USA) from healthy donors (effector:target ratio 50:1) in 200 μl RPMI 1640 complete medium (Gibco, Waltham, MA, USA) in triplicate 96-well U-bottomed plates in the presence of saturating concentrations of trastuzumab (4 μg/ml). NKG2D (1 μg/ml; R&D Systems) was used in blocking experiments. Radioactivity of the supernatant was measured with a Trilux Beta Scintillation Counter (Perkin-Elmer). Percent specific lysis was calculated as: 100 × (experimental cpm – spontaneous cpm)/(maximum cpm – spontaneous cpm).

### ELISA assay

Soluble MICA and ULBP2 were quantified in breast carcinoma cell supernatants using DuoSet ELISA kits (R&D Systems), following the manufacturer's instructions. Cell culture supernatants were collected at different time points after treatment with docetaxel and stored at −80°C until use. ELISA detection was performed using 100 μl of each supernatant. Soluble ligand quantification was normalized to the number of cells still present in culture as assessed by SRB colorimetric assay.

### Statistical analysis

Analyses were performed using GraphPad Prism 5 (GraphPad Software). Differences between groups were determined by two-tailed Student's *t*-test. Differences were considered significant at *p* < 0.05.

## SUPPLEMENTARY FIGURES


