# Supplementary material for: Taxanes enhance trastuzumab-mediated ADCC on tumor cells through NKG2D-mediated NK cell recognition
Source: Oncotarget. 2015 Nov 21;7(1):255–65. doi: 10.18632/oncotarget.6353 (PMC4807996; doi:10.18632/oncotarget.6353)
Supplement: Supplementary file 1 [file oncotarget-07-0255-s001.pdf]

SUPPLEMENTARY FIGURES

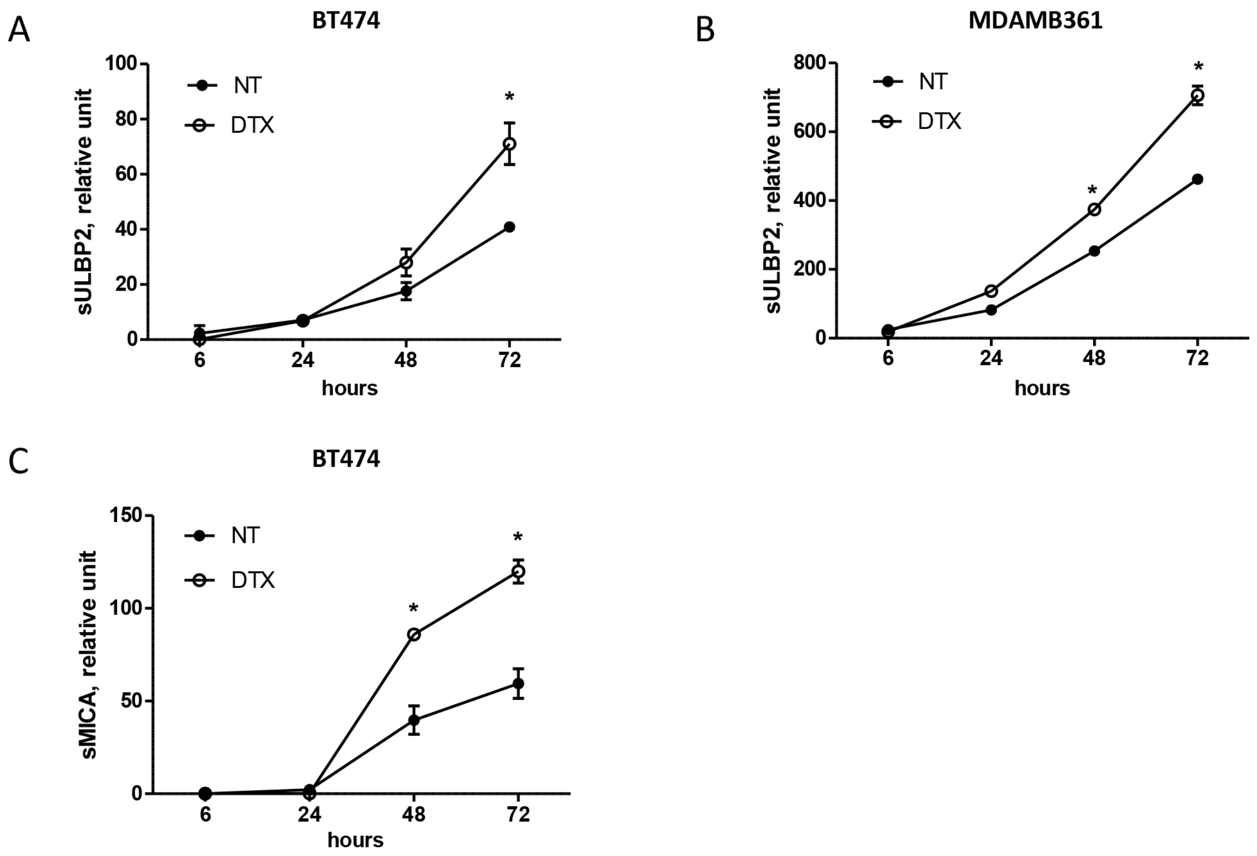

Supplementary Figure S1: ELISA evaluation of soluble ligand levels in culture supernatants of docetaxel-treated tumor cells. Soluble ULBP2 **A, B.** and MICA **C.** were evaluated in culture supernatants of BT474 (A, C) and MDAMB361 (B) cancer cells not treated (NT) or docetaxel (DTX)-treated at different time points. Data, given as mean ± SEM (n = 2), were normalized on the number of cells in culture at the time of supernatant harvesting. *$p < 0.05$ by unpaired Student's *t*-test.

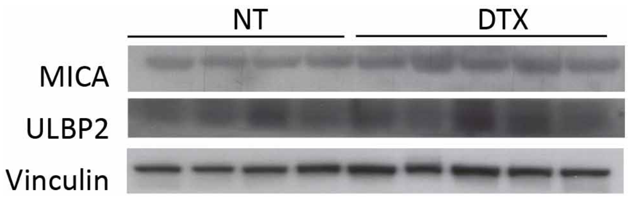

Supplementary Figure S2: Western blot analysis of NKG2D ligands MICA and ULBP2 in MDAMB361 tumors. MDAMB361 cells were xenotransplanted in SCID mice and when tumors reached a volume of 200 mm^3, mice were treated with docetaxel (DTX) at doses of 20 mg/Kg. At 24 hours after treatment, tumors were analyzed for expression of ADCC-associated factors.

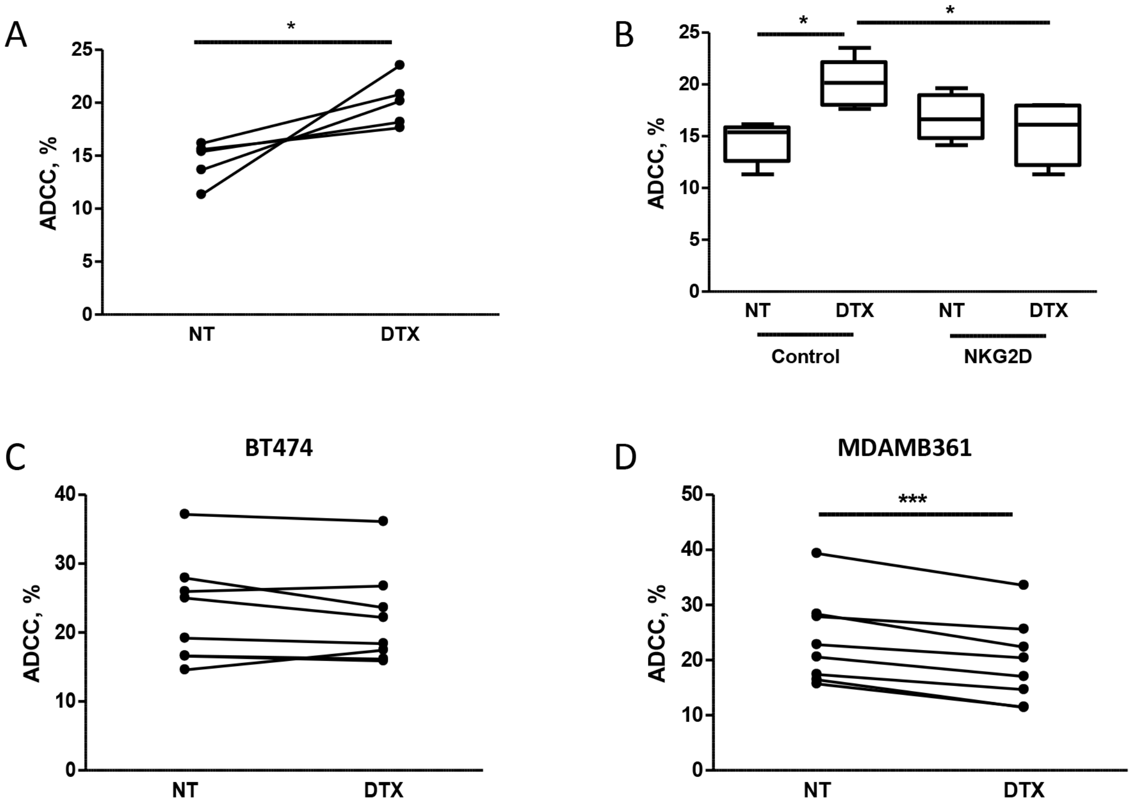

Supplementary Figure S3: ADCC of BT474 and MDAMB361 cells treated with docetaxel at different time points. A. BT474 cells treated or not with 100 nM docetaxel (DTX) for 12 hours were used in trastuzumab-mediated ADCC assay with PBMC from healthy donors as effector cells ($n = 5$). **B.** Percentage of ADCC of BT474 treated as in A, in the presence or absence of NKG2D blocking antibodies. **C, D.** Percentage of ADCC of BT474 (C) and MDAMB361 **(D)** cells treated or not with 100 nM docetaxel for 72 hours is shown. *$p < 0.05$, ***$p < 0.001$ by paired Student's *t*-test.

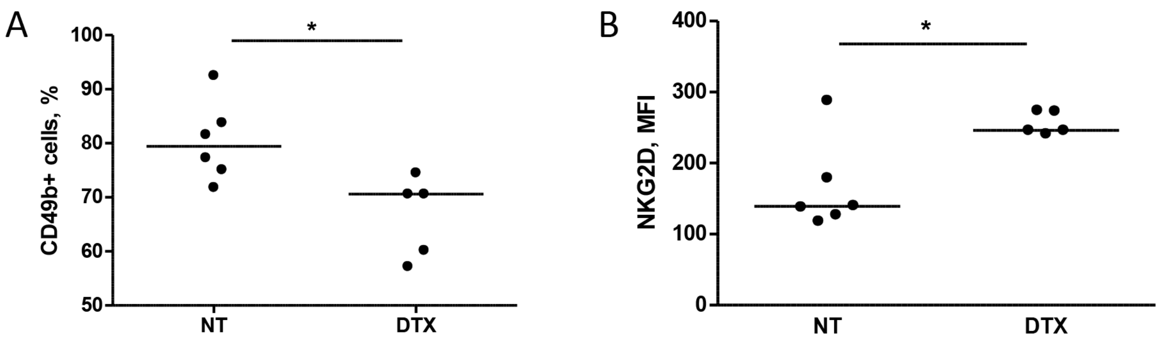

Supplementary Figure S4: Phenotype of circulating NK cells in mice bearing MDAMB361 tumors treated or not with docetaxel. A, B. Analysis of the percentage of CD49b+ cells (A) and NKG2D expression of NK cells (MFI, B) in blood of mice treated or not with docetaxel. *$p < 0.05$ by unpaired Student's *t*-test.

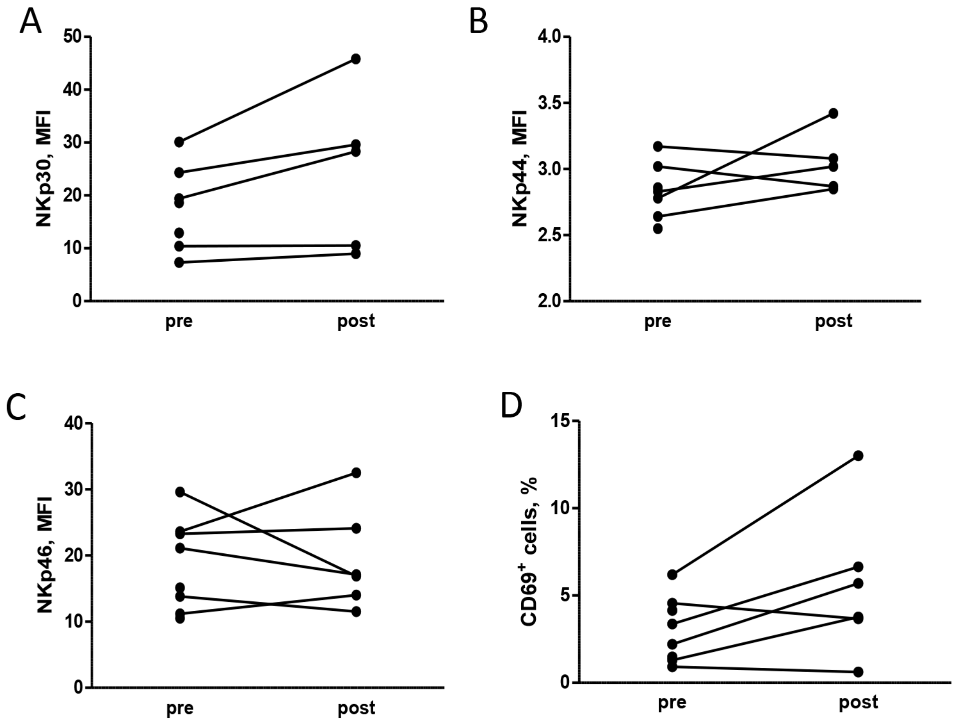

Supplementary Figure S5: Modulation of activating receptors on NK cells by chemotherapy in human patients. A, B, C. PBMC isolated from patients at different time point during neoadjuvant treatment (pre: before any treatment, post: after chemotherapy) were analyzed by flow cytometry for expression of NKp30 **(A)** NKp44 **(B)** NKp46 **(C)** receptors on CD3– CD56+ NK cells. Data are shown as MFI in each patient. **D.** Percent of $CD69^+$ cells in NK cells.

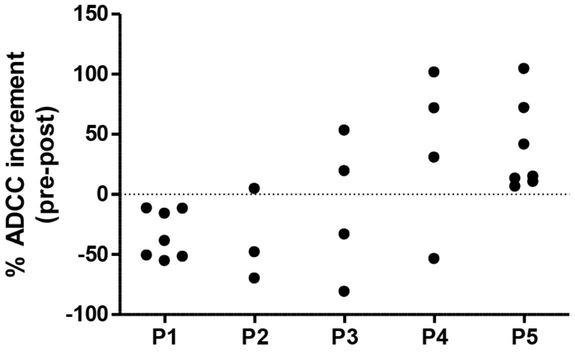

Supplementary Figure S6: ADCC increment induced by healthy donor PBMCs conditioned with pre- and post-treatment plasma from patients.
